# A logistic regression-based nomogram model incorporating clinical, dietary, and nutritional data for predicting postoperative prognosis in elderly patients of grade A tertiary hospital

**DOI:** 10.3389/fnut.2025.1644583

**Published:** 2025-10-27

**Authors:** Yi-Qiang An, Jing Wei, Na Meng, Yan-Yan Xu, Zhi-Wen Li, Shi-Hong Zhao, Wen Tong

**Affiliations:** ^1^The Health Policy Research Institute of Xuzhou Medical University, Xuzhou, Jiangsu, China; ^2^Department of Thoracic Surgery, Southeast University Affiliated Xuzhou Central Hospital, Xuzhou, Jiangsu, China; ^3^Aging Studies Institute of Xuzhou Medical University, Xuzhou, Jiangsu, China

**Keywords:** thoracic surgery, postoperative malnutrition, grade A tertiary hospital, elderly patients, dietary diversity, nutritional status, nomogram prediction model

## Abstract

**Objective:**

To develop and validate a logistic regression model predicting postoperative malnutrition risk in elderly patients using clinical, dietary, and nutritional data.

**Methods:**

We analyzed 241 elderly patients (lung cancer lobectomy/esophageal cancer resection) admitted from January 2024 to December 2024. Participants were randomized 7:3 into training (*n* = 168) and validation (*n* = 73) sets. Prognostic factors were identified via univariate analysis and multivariate logistic regression to build a predictive model. Performance was assessed using C-index, calibration curves, and receiver operating characteristic (ROC) analysis.

**Results:**

Baseline characteristics were comparable between sets (*P* > 0.05). Multivariate analysis identified number of daily food types, cereal intake, high-quality protein intake, body mass index, serum albumin, and pre-albumin as malnutrition predictors (all *P* < 0.05). The model achieved C-indices of 0.834 (training set) and 0.703 (validation set). The area under the ROC curves were 0.834 (95% CI: 0.760–0.908) and 0.703 (95% CI: 0.539–0.866), respectively, with good calibration curve fit.

**Conclusion:**

This validated model effectively predicts postoperative malnutrition risk in elderly surgical patients. Its visualization tools simplify complex nutritional assessment, offering a practical solution for resource-limited settings to improve postoperative care in grade A tertiary hospitals.

## Introduction

Lung cancer and esophageal cancer are common malignant tumors in thoracic surgery. Radical surgeries (such as lobectomy and radical resection of esophageal cancer) are the main treatment modalities. However, the incidence of postoperative malnutrition is as high as 30%−50%, which seriously affects patients' recovery, increases the risk of complications (such as anastomotic leakage and pulmonary infection), and prolongs the length of hospital stay (1). In elderly patients, the risk of malnutrition is further elevated due to factors such as decreased metabolic function, surgical trauma stress, and postoperative eating disorders (such as reduced intake caused by digestive tract reconstruction after esophageal cancer surgery and impaired appetite due to cough and expectoration after lung cancer surgery) (2). Currently, clinical nutritional assessment relies on subjective indicators (such as body weight and dietary questionnaires) or single laboratory indicators (such as serum albumin), lacking a multi-dimensional assessment tool that integrates clinical features, dietary structure, and objective nutritional indicators. Studies have shown that insufficient dietary diversity (few daily food types), insufficient intake of energy and high-quality protein were directly related to postoperative malnutrition, while BMI, serum albumin, and pre-albumin are key indicators reflecting the body's nutritional reserve. The unique pathophysiological changes in thoracic surgery (such as decreased digestive and absorption functions caused by gastric replacement of the esophagus after esophageal cancer surgery and impaired body metabolism due to damaged lung function after lung cancer surgery) further exacerbate the complexity of nutritional risk (3). In this study, based on a postoperative cohort of elderly patients in grade A tertiary hospitals, logistic regression methods were used to integrate clinical features (surgical type, comorbidities), dietary parameters (food types, nutrient intake), and objective nutritional indicators to construct a postoperative prognosis prediction model. The aim was to provide a “one-stop” assessment tool for the early clinical identification of high-risk postoperative patients (such as those with malnutrition and complications) and to improve the prognosis through precise nutritional interventions (such as early enteral nutritional support and dietary guidance after surgery). Especially, this integrated, equipment-free tool addresses the limitations of traditional assessments in resource-limited grade A tertiary hospitals, which often rely on subjective judgment or single laboratory indicators.

## Materials and methods

### Study subjects

A total of 241 elderly patients admitted to the Department of Thoracic Surgery of the hospital from January 2024 to December 2024 were included. The surgical procedures included lobectomy for lung cancer and radical resection for esophageal cancer. Inclusion criteria were as follows: (1) Patients met the diagnostic criteria for lung cancer or esophageal cancer and underwent radical surgery; (2) Patients had complete clinical data; patients had a postoperative hospital stay of ≥ 7 days; (3) Patients were aged ≥ 60 years, consistent with WHO definitions of “elderly” and reflecting the population at highest risk of postoperative malnutrition. Exclusion criteria were as follows: (1) Patients had severe malnutrition before surgery (body mass index (BMI) <16 kg/m^2^ or serum albumin <25 g/L); (2) Patients had severe liver dysfunction (Child-Pugh class B/C) or severe kidney dysfunction [estimated glomerular filtration rate (eGFR) <30 mL/min/1.73m^2^, calculated from serum creatinine levels using the Chronic Kidney Disease Epidemiology Collaboration (CKD-EPI)]; (3) Patients received radiotherapy, chemotherapy, or immunotherapy before surgery; (4) Patients developed severe postoperative complications (such as anastomotic leakage or respiratory failure), as these complications significantly confound nutritional status independent of baseline risk factors. The patients were divided into a training set (*n* = 168) and a validation set (*n* = 73) at a ratio of 7:3 using the random number table method. This study was approved by the ethics committee of Southeast University Affiliated Xuzhou Central Hospital (No. 37245), and informed consent was signed by all patients. Clinical trial number: not applicable.

### Data collection

(1) Clinical characteristics: Age, gender, type of surgery (lung cancer/esophageal cancer), comorbidities (diabetes, hypertension, coronary heart disease), operation time, post-operative hospital stay days, smoking history, and drinking history. **(**2) Dietary survey: The number of daily food types, cereal intake, and high-quality protein (fish, meat, eggs, milk) intake were recorded for three consecutive days using the 24-h dietary recall method [multiple-pass method (MPM)] administered by nurses who received special training on the standard operating procedures of this method (not professional dietitians) (4, 5). The data from three consecutive 24-h dietary recalls were averaged to obtain a more stable estimate of habitual intake and reduce day-to-day variability. **(**4) Nutritional indicators: Pre-operative BMI, serum albumin, and pre-albumin levels (measured on postoperative day 3). The above-mentioned indicators are all clinical data routinely available in grade A tertiary hospitals (such as BMI and serum protein tests) and the results of simple dietary surveys (without the participation of professional dietitians), which are suitable for large-scale promotion and application in primary medical settings. **(**5) Outcome indicators: The diagnostic criterion for malnutrition was defined as a NRS2002 score ≥ 3 points during hospitalization. This short-term outcome was selected to align with the clinical need of grade A tertiary hospital to prioritize early postoperative rehabilitation management and guide timely nutritional intervention.

### Statistical analysis

Data analysis was conducted using SPSS 25.0 and R 4.4.2 software. Using the “caret” package in R 4.4.2, the subjects were randomly divided into a training set and a validation set in a 7:3 ratio. Measurement data were expressed as mean ± standard deviation. An independent-samples t-test was used for comparisons between two groups, and analysis of variance was employed for comparisons among multiple groups. Count data were presented as the number of cases and percentages (*n*, %). The χ^2^ test was used for comparisons between groups. Univariate analysis and multivariate logistic regression analysis were used to screen independent factors affecting the treatment efficacy. The results were presented using the odds ratio (OR) and its 95% confidence interval (CI). Nomogram prediction model was used to construct the postoperative prognosis model. Evaluate the predictive performance of the model through the receiver operating characteristic (ROC) curve and calculate the area under the curve (AUC) and its 95% CI. Use calibration curves to evaluate the consistency between model predictions and actual observation, *P* < 0.05 indicates statistical significance. The “rmda” package and “dcurves” package in R language were used to draw decision curve analysis (DCA) curves to evaluate whether the model was clinically beneficial.

## Results

### Comparison of baseline characteristics of patients in the training set and the validation set

A total of 241 elderly patients, who were randomized 7:3 into training (*n* = 168) and validation (*n* = 73) sets, were admitted. No significant differences were found in indicators such as age, gender, surgical type, and comorbidities between the training set and the validation set (all *P* > 0.05), indicating comparability (Table 1).

**Table 1:** Comparison of baseline characteristics of patients in the training set and the validation set.

**Indicator**	**Training set (*n* = 168)**	**Validation set (*n* = 73)**	**χ^2^/*t***	***P***
Age (years)	84.81 ± 3.99	84.55 ± 2.02	0.527	0.598
Sex (Male/Female)	90/78 (53.57/46.42)	40/33 (54.79/45.21)	0.031	0.861
Surgery type (Lung cancer/Esophageal cancer)	105/63 (62.50/37.50)	50/23 (68.49/31.51)	0.796	0.372
Comorbid diabetes	38/130 (22.62/77.38)	10/63 (13.69/86.31)	2.538	0.111
Comorbid hypertension	80/88 (47.62/52.38)	30/43 (41.09/58.91)	0.873	0.351
Comorbid coronary heart disease	40/128 (23.81/76.19)	25/48 (34.25/65.75)	2.814	0.093
Operation time (hours)	3.51 ± 1.12	3.55 ± 1.26	0.245	0.806
Post-operative hospital stay (days)	13.12 ± 4.01	13.55 ± 4.52	0.735	0.463
Smoking history (≥10 packs/year)	84/84 (50.00/50.00)	40/33 (54.79/45.21)	0.468	0.494
Drinking history (≥5 years)	60/108 (35.71/64.29)	28/45 (38.36/61.64)	0.153	0.695
Number of daily food types (categories)	4.36 ± 1.52	4.51 ± 1.66	0.684	0.494
Cereal intake (g/d)	210.18 ± 55.11	211.56 ± 85.66	0.149	0.881
High-quality protein intake (g/d)	48.52 ± 15.38	47.66 ± 12.58	0.420	0.675
BMI (kg/m^2^)	19.91 ± 2.42	20.15 ± 2.01	0.743	0.458
Serum albumin (g/L)	35.58 ± 3.62	34.56 ± 4.52	1.859	0.064
Pre-albumin (mg/L)	189.51 ± 38.42	192.65 ± 40.66	0.573	0.567

### Univariate analysis of risk factors for postoperative prognosis in elderly patients from grade A tertiary hospitals in the training set

In the training set, patients were divided into the poor prognosis group (*n* = 58) and the good prognosis group (*n* = 110). The result of univariate analysis showed that there were significant differences in indicators such as surgery type, comorbid diabetes, number of daily food types, cereal intake, and high-quality protein intake, BMI, serum albumin and pre-albumin (all *P* < 0.05; Table 2).

**Table 2:** Univariate analysis of risk factors for postoperative prognosis in elderly patients from grade A tertiary hospitals in the training set.

**Indicator**	**Poor prognosis group (*n* = 58)**	**Good prognosis group (*n* = 110)**	**χ^2^/*t***	***P***
Age (years)	65.01 ± 3.90	64.42 ± 3.50	0.998	0.319
Sex (Male/Female)	36/22 (62.07/37.93)	54/56 (59.09/50.91)	2.571	0.108
Surgery type (Lung cancer/Esophageal cancer)	16/42 (27.59/72.41)	89/21 (80.90/19.10)	46.071	0.001
Comorbid diabetes	26/32 (44.83/55.17)	12/98 (10.91/89.09)	24.961	0.001
Comorbid hypertension	32/26 (55.17/44.83)	48/62 (43.63/56.37)	2.026	0.154
Comorbid coronary heart disease	18/40 (31.03/68.97)	22/88 (20.00/80.00)	2.549	0.111
Operation time (hours)	3.61 ± 1.22	3.43 ± 0.91	1.081	0.282
Post-operative hospital stay (days)	13.52 ± 4.21	12.81 ± 3.12	1.239	0.217
Smoking history (≥10 packs/year)	34/24 (58.62/41.38)	50/60 (45.45/54.55)	2.633	0.105
Drinking history (≥5 years)	22/36 (37.93/62.07)	38/72 (34.54/65.46)	0.189	0.663
Number of daily food types (categories)	3.86 ± 1.12	4.51 ± 1.32	3.192	0.002
Cereal intake (g/d)	190.12 ± 50.12	220.65 ± 60.85	3.278	0.001
High-quality protein intake (g/d)	45.12 ± 15.35	50.65 ± 15.44	2.211	0.028
BMI (kg/m^2^)	19.81 ± 2.12	20.88 ± 2.55	2.735	0.007
Serum albumin (g/L)	34.58 ± 3.52	36.12 ± 3.83	2.567	0.012
Pre-albumin (mg/L)	180.56 ± 35.22	200.18 ± 40.36	3.126	0.002

### Multivariate logistic regression analysis

The prognostic effect is the dependent variable (poor prognosis = 1, good prognosis = 0), and variables with *P* < 0.05 in univariate analysis are used as covariates. The results of multiple logistic regression analysis showed that the higher the number of daily food types, grains, and high-quality protein, the higher the BMI, and the higher the serum albumin and pre-albumin levels were associated with the lower the risk of postoperative malnutrition in elderly patients (all *P* < 0.05; Table 3).

**Table 3:** Results of multivariate logistic regression analysis.

**Indicator**	***B***	***SE***	***Wald***	***P***	***OR***	**95% *CI***
Number of daily food types	−0.532	0.155	11.841	0.001	0.587	0.434–0.795
Cereal intake	−0.010	0.004	7.857	0.005	0.990	0.983–0.997
High-quality protein intake	−0.041	0.014	9.107	0.003	0.960	0.934–0.986
BMI	−0.265	0.085	9.662	0.002	0.767	0.649–0.907
Serum albumin	−0.157	0.057	7.439	0.006	0.855	0.764–0.957
Pre-albumin	−0.012	0.005	4.837	0.028	0.988	0.978–0.999

### Construction of a nomogram prediction model

**Figure 1:**
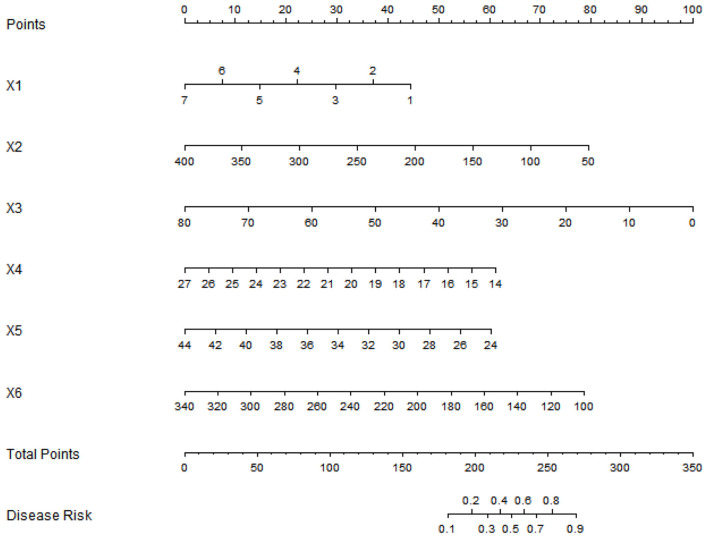
Nomogram of prognostic model for elderly postoperative patients (X1: Number of daily food types, X2: Cereal intake, X3: High-quality protein intake, X4: BMI, X5: Serum albumin, X6: Pre-albumin).

Based on multiple logistic regression analysis, the influencing factors of postoperative prognosis in elderly patients were visualized using a column chart. Assign scores to each factor in the model and calculate the total score for predicting clinical efficacy, represented by prediction probability (Figure 1).

### Evaluation and validation of the nomogram prediction model

**Figure 2:**
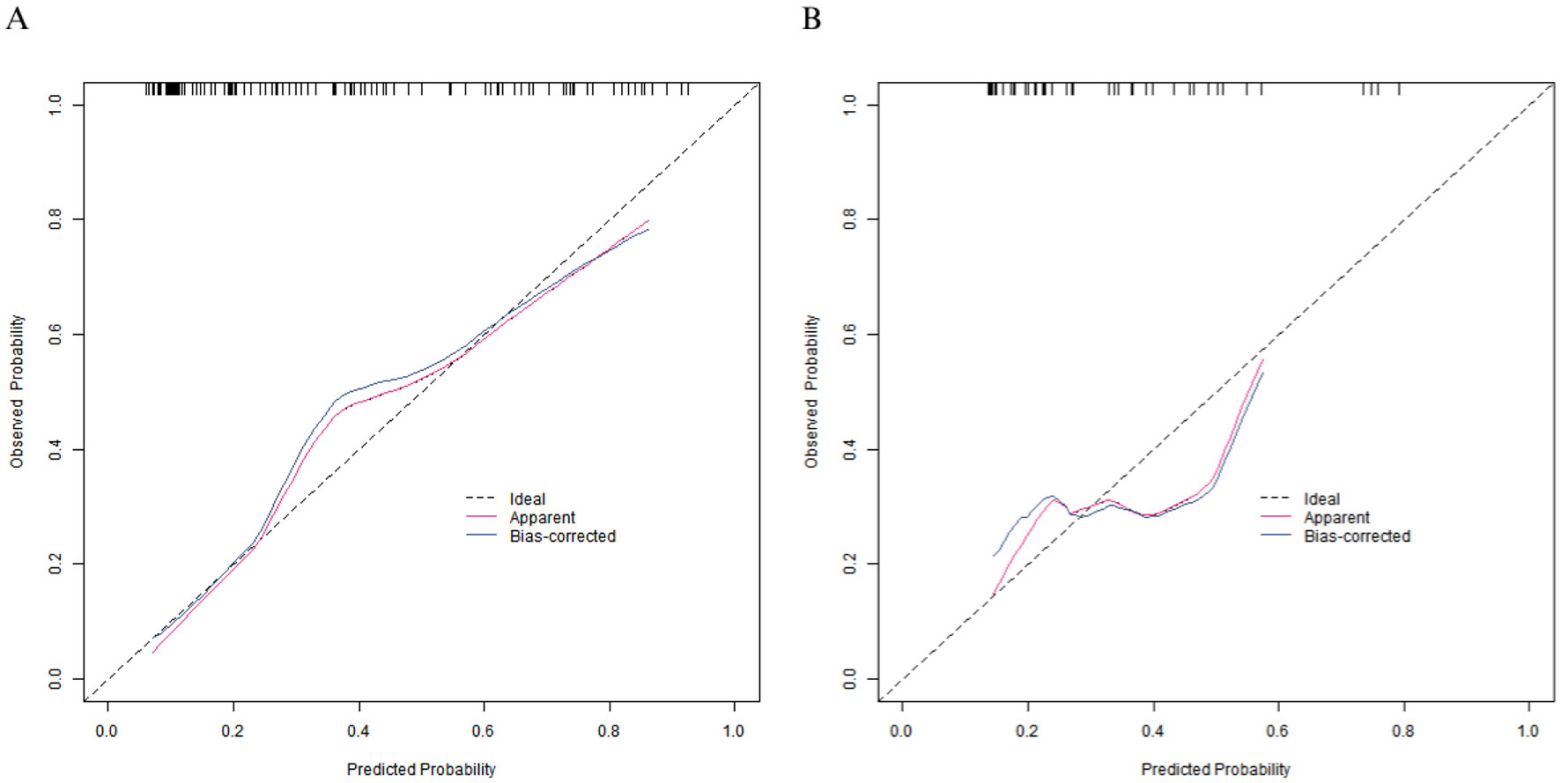
Calibration curves in the training set **(A)** and the validation set **(B)**.

**Figure 3:**
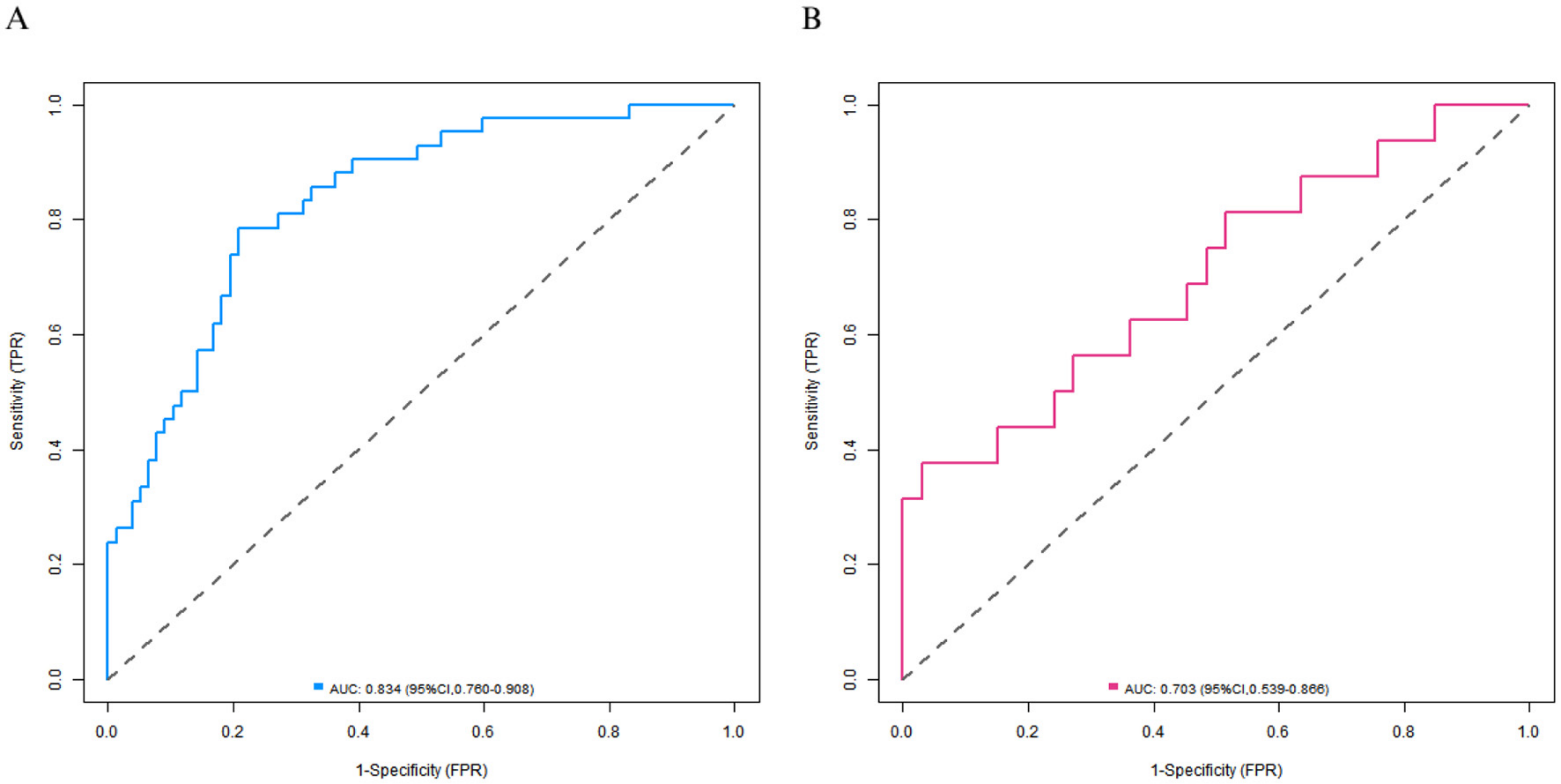
Receiver operating characteristic curves in the training set **(A)** and the validation set **(B)**.

In the training set, the C-index of the calibration curve was 0.834 and the Brier score was 0.158. The *P-value* obtained from Hosmer-Lemeshow test was 0.604, indicating a good fit of the model. The ROC curve showed an AUC of 0.834 (95% CI: 0.760–0.908), with a sensitivity of 0.786 and a specificity of 0.792. In the validation set, the C-index was 0.703, the Brier score was 0.193, the *P-value* of Hosmer Lemeshow test was 0.349, the area under the ROC curve was 0.703 (95% CI: 0.539–0.866), and the sensitivity and specificity were 0.562 and 0.727, respectively. The calibration curve and the ROC curve are shown in Figures 2, 3.

### Decision curve analysis

**Figure 4:**
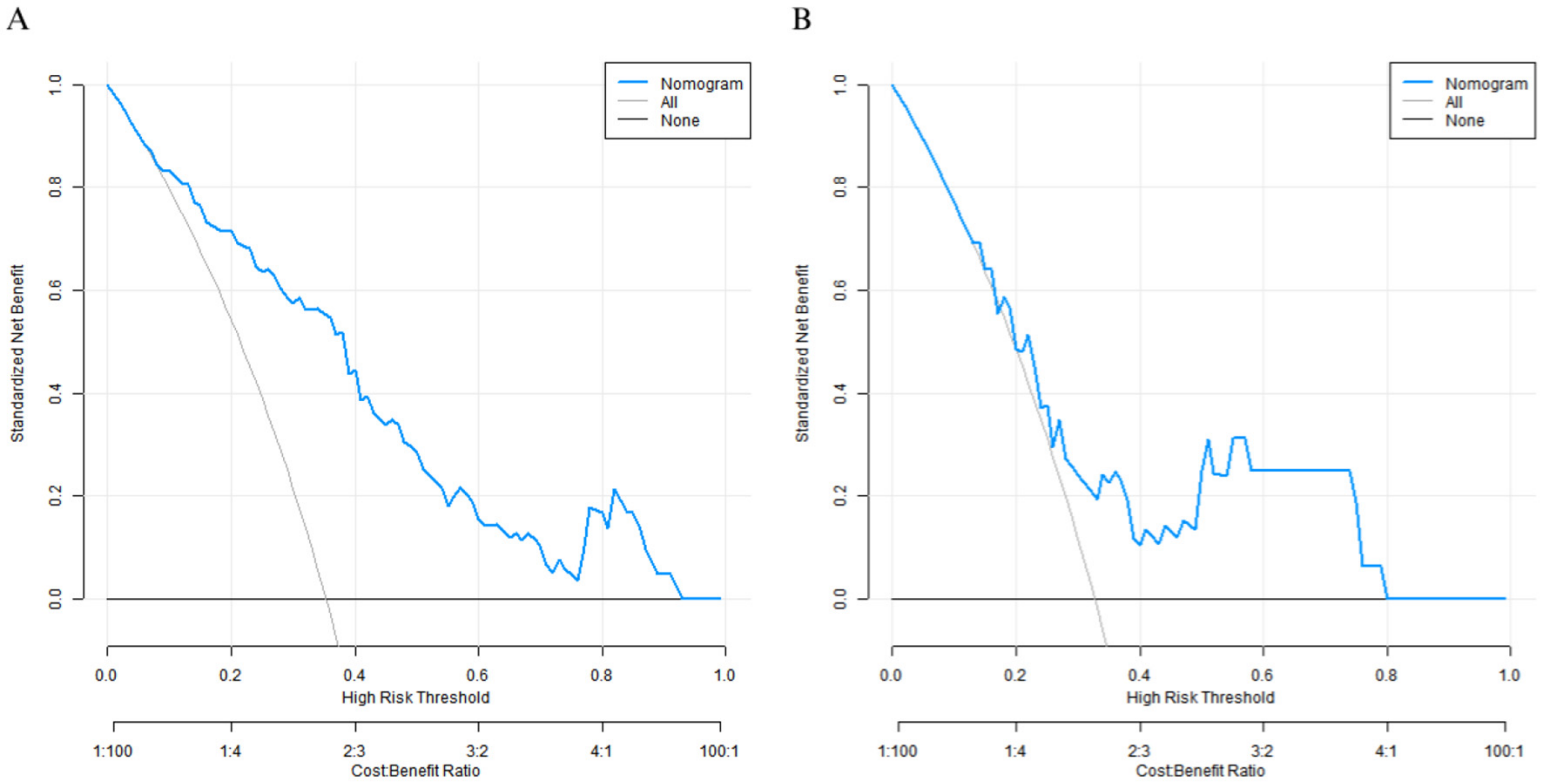
Decision curves in the training set **(A)** and the validation set **(B)**.

Decision curve analysis showed that when the threshold probability was between 0.10–0.80, the decision to predict the nutritional status of elderly patients' care using the nomogram prediction model for good prognosis of elderly patients after surgery in this study had the optimal net benefit (Figure 4).

## Discussion

Based on the data of 241 elderly patients after thoracic surgery, this study screened out 6 influencing factor risk factors (daily number of food types, cereal intake, high-quality protein intake, BMI, serum albumin, pre-albumin) through univariate and multivariate Logistic regression and constructed a nomogram prediction model. The model showed good predictive performance in both the training set and the validation set: the C-index in the training set was 0.837, and the AUC was 0.834; the C-index in the validation set was 0.703, and the AUC was 0.703. The calibration curve showed a good fit between the predicted values and the actual values.

Although univariate analysis linked surgical type (lung cancer vs. esophageal cancer) and diabetes to malnutrition, these factors were not retained in the multivariate model. This suggests that their impact on nutritional status may be mediated through the dietary and nutritional indicators included in the final model, such as food variety and protein intake (6). This suggests that the direct impact of clinical features may be indirectly reflected through dietary and nutritional status, highlighting the necessity of integrating multi-dimensional indicators (7). The daily number of food types is an important predictor, reflecting the direct association between insufficient dietary diversity and malnutrition (8). Elderly patients often have a single intake due to difficulties in chewing, changes in taste, etc., leading to a lack of essential nutrients. Especially, patients after esophageal cancer surgery are more likely to have dietary restrictions due to gastrointestinal reconstruction. BMI, serum albumin, and pre-albumin, as classic nutritional indicators, respectively reflect the short-term and long-term nutritional status (9). Pre-albumin has a short half-life (about 2 days) and can sensitively reflect acute malnutrition, while serum albumin has a long half-life (about 21 days), reflecting long-term nutritional reserve. The combination of the two can dynamically evaluate nutritional changes (10). The model's feasibility in primary care is high, as it utilizes routine laboratory measures (BMI, albumin, pre-albumin) and simple dietary data obtainable by nurses without specialized equipment or a nutrition team. Through the visual scoring design, the nomogram transforms the complex multi-factor analysis into an intuitive calculation of risk probability. Primary medical staff do not need to be proficient in machine learning algorithms and can complete the assessment by simply substituting the patients' basic data, which reduces the technical threshold and meets the needs of primary healthcare for simplified tools. Decision curve analysis shows that when the threshold probability is between 0.10–0.80, the model has the best net benefit, indicating its suitability for most clinical scenarios. Compared with traditional single indicators, the nomogram integrates the “cause-intake-reserve” chain (for example, surgical type affects intake, and insufficient intake leads to a decline in nutritional reserve), which is more in line with the pathophysiological characteristics of elderly patients after thoracic surgery. The validation set's performance (C-index 0.703) was lower than the training set's (0.834), potentially due to the smaller sample size (*n* = 73) and minor baseline differences (e.g., serum albumin, *P* = 0.064). However, the calibration curve still shows good consistency, indicating that the model has certain generalization ability. In the future, it can be further optimized by expanding the sample size or conducting multi-center studies.

Considering that elderly patients in primary care often have a single dietary structure due to factors such as economic conditions and eating habits, the model sets easily intervenable indicators such as daily number of food types and high-quality protein intake as core variables, which facilitates primary medical staff to quickly improve patients' nutritional status through targeted dietary guidance (such as recommending local seasonal ingredients and family-style nutritional supplements), reflecting the local adaptation intervention strategy.

As the core indicator of dietary diversity, it is negatively correlated with malnutrition (OR = 0.587). The World Health Organization (WHO) recommends consuming at least 12 different food types daily to ensure adequate dietary diversity and balanced nutrient intake. However, the average number of food types consumed by patients in this study was only 4.36, significantly lower than this recommendation (11).

Elderly patients often simplify their diet after surgery due to weakened digestive function and psychological factors (such as fear of choking), leading to a lack of micronutrients such as vitamins and minerals, which affects immune function and tissue repair (12). Cereals are the main source of carbohydrates, providing basic energy. This study shows that insufficient cereal intake (for every 1g increase, OR decreases by 0.990) is related to malnutrition, possibly because insufficient energy intake leads to enhanced catabolism (13). Patients after esophageal cancer surgery are particularly prone to limited cereal intake due to the reduced gastric capacity caused by gastric replacement of the esophagus, resulting in early satiety (14).

High-quality protein (fish, meat, eggs, milk) is a high-quality source of amino acids, directly affecting postoperative tissue repair and immunoglobulin synthesis. Elderly patients generally have a vicious cycle of inflammation-sarcopenia. Insufficient protein intake (for every 1g increase, OR decreases by 0.960) exacerbates muscle breakdown, leading to a decline in immunity and an increased risk of anastomotic leakage.

Insufficient reserve is the core manifestation. As an indicator reflecting the overall nutritional status, a lower BMI (OR = 0.767) indicates long-term insufficient nutritional reserve. The preoperative BMI of elderly patients is often affected by chronic diseases (such as progressive dysphagia caused by esophageal cancer). After surgery, the energy consumption increases under the stress state (10%−30% higher than the basal metabolic rate), exacerbating the negative nitrogen balance (15).

The gold standard for traditional nutritional assessment, its decrease (OR = 0.855) is closely related to postoperative complications (16). When serum albumin is <35g/L, the wound-healing ability decreases, and the risk of infection increases. However, it should be noted that it is affected by liver synthesis function and inflammatory status, and may show pseudo-normal in the early postoperative stage due to the stress response, so it is necessary to dynamically evaluate it in combination with pre-albumin (17).

As a sensitive indicator of visceral protein, it has a short half-life and can quickly reflect recent nutritional changes. In this study, a decrease in pre-albumin (OR = 0.988) is an independent risk factor, especially suitable for monitoring the effect of early postoperative nutritional intervention, such as whether enteral nutritional support can effectively improve the short-term nutritional status.

Dietary indicators and nutritional status indicators are in a chain-like association: insufficient dietary diversity and protein intake directly lead to a lack of energy and nutrients, which in turn affects the levels of BMI (long-term) and serum albumin and pre-albumin (short-term) (18). The trauma stress of thoracic surgery further amplifies this effect: the increase in cytokines (such as IL-6, TNF-α) caused by surgery inhibits liver albumin synthesis and accelerates muscle breakdown. Even with sufficient intake, the nutritional indicators may still decline. Therefore, the indicators in the model do not exist in isolation but jointly reflect the dynamic imbalance of intake-metabolism-reserve, which is also the scientific basis for multi-dimensional assessment.

This study has several limitations. Firstly, the lack of external validation with an independent cohort from a different institution and the model was developed and validated on a single-institution dataset, which may introduce bias and limit its generalizability. The study subjects are from a single hospital. Although the baseline data show that the training set and the validation set are balanced, regional differences in dietary habits (e.g., staple foods, protein sources), surgical protocols, and nutritional support practices may affect the universality of the model. Future multi-center external validation studies are essential to assess the model's robustness, adjust for geographic and institutional variations, and confirm its utility across diverse healthcare settings. Secondly, due to the limited number of cases included during the study period (241 cases) and the complex comorbidities of elderly patients after thoracic surgery, the sample size required for external validation is large, and it is difficult to complete in the short term. The relatively small validation set (*n* = 73) may limit the precision of performance metrics, as reflected by wider confidence intervals; this is mainly due to the single-center nature of the study and the limited number of eligible elderly thoracic surgery patients during the study period (January-December 2024). Future studies should adopt k-fold cross-validation for internal validation and expand to multi-center external validation with larger cohorts to enhance generalizability. Thirdly, assessing nutritional status at a single early time point may be influenced by transient surgical stress. Future studies are recommended to conduct repeat assessments at 5–7 days postoperatively to better differentiate transient changes from true nutritional decline. The outcome indicator was malnutrition during the postoperative hospital stay (NRS2002 score), lacking long-term follow-up data on nutritional status trajectories and prognosis after discharge. This limits understanding of the model's relevance to sustained malnutrition. Notably, the model's structure—incorporating dynamic dietary and laboratory markers—could be adapted for longitudinal prediction by integrating measurements at later time points (e.g., 1-month post-discharge). The follow-up period should be extended in future studies to explore this. Fourthly, postoperative inflammatory indicators (such as C-reactive protein), body composition analysis (such as muscle mass), and comprehensive comorbidity indices are not included. These indicators may enhance predictive accuracy but were excluded because they are not routinely measured in resource-limited grade A tertiary hospitals. Future iterations could incorporate them if they become more accessible in primary care settings, and multi-center studies with expanded data collection capacity are needed to explore their utility. In addition, we excluded patients who experienced severe postoperative complications (e.g., anastomotic leakage, respiratory failure). While this exclusion was necessary to minimize confounding and establish a clear predictive relationship between preoperative/near-postoperative nutritional risk and outcomes without the profound pathophysiological changes associated with major morbidity, it consequently limits the generalizability of our model to this most vulnerable and complex patient subgroup. We recognize that these patients are at the highest risk for malnutrition, and future research should specifically focus on validating existing models or developing new ones tailored to predict nutritional risk in the context of major postoperative complications. Future studies with larger sample sizes should aim to validate and potentially refine nutritional risk prediction models separately for distinct surgical types, such as lung and esophageal resections, to account for their unique physiological impacts and recovery trajectories.

Combine the changes in nutritional indicators at different postoperative time points (such as 3 days and 7 days after surgery) to construct a dynamic prediction model and guide staged nutritional intervention. Based on the high-risk patients identified by the nomogram, conduct an efficacy evaluation of individualized nutritional support (such as early enteral nutrition and oral nutritional supplements) to form a closed-loop of assessment-intervention-monitoring. In primary care, this model could be integrated into the electronic medical record to automatically capture patient data and generate a risk score. This would embed nutritional risk assessment into the routine postoperative workflow, creating a closed-loop system for early screening, intervention, and monitoring. This lightweight tool design is highly compatible with the information construction level of grade A tertiary hospitals, which helps to improve the overall diagnosis and treatment efficiency. While this study prioritizes an interpretable logistic regression model suitable for grade A tertiary hospitals, future research with larger, multi-center datasets could explore more complex algorithms like deep learning, provided clinical interpretability is maintained.

In conclusion, the nomogram prediction model constructed in this study integrates the dietary diversity, nutritional status, and clinically relevant indicators of elderly patients after thoracic surgery, providing visualization tool for the assessment of the risk of malnutrition. The key indicators in the model not only reflect nutritional intake and reserve but also reflect the impact of surgical trauma on nutritional metabolism, which is in line with the complex pathophysiological characteristics of elderly patients.

## Data Availability

The raw data supporting the conclusions of this article will be made available by the authors, without undue reservation.
